# Efficacy and adverse effects of oral chelating therapy (deferasirox) in multi-transfused Pakistani children with β-thalassemia major

**DOI:** 10.12669/pjms.313.6972

**Published:** 2015

**Authors:** Muzamil Shabana Ejaz, Shagufta Baloch, Fehmina Arif

**Affiliations:** 1 Dr. Muzamil Shabana Ejaz, Associate Professor, Department of Pediatrics Dow University of Health Sciences Civil Hospital Karachi, Karachi - Pakistan; 2 Dr. Shagufta Baloch, FCPS Trainee, Department of Pediatrics Dow University of Health Sciences Civil Hospital Karachi, Karachi - Pakistan; 3 Prof. Fehmina Arif, Professor, Department of Pediatrics Dow University of Health Sciences Civil Hospital Karachi, Karachi - Pakistan

**Keywords:** Adverse effects, Children, Deferasirox, Efficacy, Oralchelation, Thalassemia

## Abstract

**Objective:**

To determine the efficacy and adverse effects of oral chelation therapy (deferasirox) in multi-transfused β-thalassemia major patients visiting pediatric thalassemia clinic in Civil Hospital Karachi.

**Methods:**

This prospective study was conducted at pediatric thalassemia clinic of Civil Hospital Karachi. Hundred multi-transfused β-thalassemia patients registered in the clinic for oral iron chelation therapy were included in the study. Information regarding clinical and laboratory parameters including abdominal pain, jaundice, serum ferritin, creatinine and serum transaminase levels were recorded on a Performa and data was analyzed through SPSS 16.

**Results:**

Hundred patients were stratified into two age groups, 54% were below and 46% were above nine year. Majority were males, 62% and 38% were females. Abdominal pain 41%, nausea 31%, vomiting 15%, jaundice 15% and elevated serum creatinine 11.5% were frequently observed clinical adverse effects in this study. Serum glutamic pyruvic transaminase (SGPT) level was statistically significant compared with initial visit and six months after optimal chelation therapy (p=0.030). Although Serum ferritin was decreased but it was not statistically significant (p=0.929).

**Conclusion:**

Deferasirox is an effective oral chelation agent for β-thalassemia major patients. Most common adverse effects of the drug are abdominal pain, nausea, vomiting, and elevation of liver enzymes.

## INTRODUCTION

β-thalassemia is one of the major health problems in Pakistan with a carrier rate of 5-8%.^1^ Thalassemia major patients require regular blood transfusions which in turn lead to secondary iron overload.^2^ They need lifelong chelation therapy to prevent complications associated with chronic iron over load including liver and cardiac diseases, retardation of growth and sexual development during adolescence.^3^,^4^

Multiple iron chelating drugs with various regimens and mode of administration are used in transfusion dependent β-thalassemia major patients including parenteral deferoxamine, oral deferiprone and deferasirox. These chelating agents showed variable efficacy and side effects.^5^ Initial chelation therapy with deferoxamine was associated with poor compliance due to slow parenteral infusions and poor long-term outcome therefore oral chelating drugs deferasirox and deferiprone were introduced to prevent complications of iron over load, which showed improved compliance and efficacy.^6^ However side effects including arthritis, 5-20%, neutropenia, 5%, and severe agranulocytosis, 0.5%, were observed in patients treated with deferiprone which limited its use.^7^ Deferasirox provides an effective alternative to deferiprone and deferoxamine in multi-transfused patients especially with intolerance and poor compliance to these drugs.^8^ It is recommended as once daily dose in children more than two year of age.^9^,^10^ Deferasirox have high iron binding capacity and selectivity.^11^ It can reduce serum ferritin levels in dose dependent manner and is relatively a safe drug but treatment requires individualization with careful dose adjustment and proper monitoring.^9^,^10^ Apart from reduction in major parameters including serum ferritin, liver iron concentration and toxic labile plasma iron, deferasirox has also reported to decrease and prevent cardiac iron overload with continuous long term therapy.^12^

Studies have shown that deferasirox stabilizes serum ferritin and liver iron concentrations when used in a dose of 20mg/kg/day whereas it significantly reduces these indicators and leads to negative iron balance in transfusion dependent patients in a dose of 30-40mg/kg/day.^13^ Side effects of deferasirox are transient and mild to moderate in intensity. Most common side effects reported are gastrointestinal disturbances (16.6%), increased blood creatinine (11.2%), skin rashes (6.6%) and reversible cytopenias. Elevation of serum liver transaminases is transient and reduced in frequency over time.^3^,^9^ Pediatric growth and adolescent sexual development were not affected.^3^ As the transfusion and iron chelating therapy is lifelong so there is need to assess the long-term effects of deferasirox.

Deferasirox is being used in multi-transfused β-thalassemia patients of civil hospital Karachi and previously no study was done to assess the efficacy and side effects of this drug in our setup therefore this study was done to assess the effects of deferasirox in these patients in order to provide better drug monitoring and outcome which can ultimately improve the patients compliance and their quality of life. This study was done to determine the efficacy and adverse effects of oral chelation therapy (Deferasirox) in multi-transfused β-thalassemia major patients visiting pediatric thalassemia clinic in Civil Hospital Karachi.

## METHODS

This is a hospital based prospective study conducted at thalassemia clinic of Pediatric Unit-II of Dow University of Health Sciences and Civil Hospital Karachi after approval from the Ethical review board from May 2012 to April 2013 for duration of one year. This is a tertiary care hospital and caters urban and periurban population of Sindh province in Pakistan.

The study included hundred multi-transfused β-thalassemia patients between two to sixteen year of age through convenient sampling. These patients were registered in thalassemia clinic for at least one year and had received optimal oral chelation with deferasirox, 20-40 mg/kg/day for six months or above. Patients with serum ferritin level more than 1000 ng/ml, receiving repeated transfusions were included in the study. Children with multiple transfusions and receiving chelation therapy due to other causes like sickle cell anemia, osteopetrosis, hereditary spherocytosis and auto immune hemolytic anemia were excluded from the study. Β-thalassemia patients suffering from severe acute illnesses including congestive cardiac failure, renal diseases, bronchopneumonia, hepatitis and deranged liver enzymes at the beginning were also excluded from the study.

All the patients were evaluated through a performa filled by co-investigator and a resident medical officer after informed consent from patient or attendant. These patients were assessed for efficacy and possible adverse effects, nausea, vomiting, abdominal pain, jaundice through history and physical examination. Laboratory investigations including complete blood picture, serum ferritin, creatinine and transaminase levels were done at the initial visit and then repeated after every three months of initiation of deferasirox from Central laboratory of Civil Hospital Karachi.

Data analyses were done by using SPSS version 16. Mean and Standard Deviation was calculated for quantitative variables like age and laboratory parameters. Qualitative variables like gender, ethnicity, clinical features, nausea, vomiting, jaundice and anemia were presented as frequencies and percentages. The initial laboratory tests were compared after six months by stratification and applying Paired Sample test for quantitative variables.

## RESULTS

In this study hundred Thalassemia major children were included. These patients were stratified in to two age groups 54% were below and 46% were above nine year of age. There were 62% males and 38% females. Regarding ethnicity majority were Sindhi, 36%, Pathans 19%, Baloch, 17%, Punjabi, 3% whereas other ethnic groups accounted for 25%.

The frequency of clinical adverse effects shows that anemia was present in 70%, abdominal pain 41%, nausea 31%, vomiting and jaundice was present in 15% patients, respectively. Clinical efficacy was assessed by stratification of serum ferritin, creatinine and serum glutamic pyruvic transaminase (SGPT) values and further evaluated by paired sample test. Serum ferritin values when compared with initial visit and six months after the optimal chelation therapy showed P value of (P=0.929). Haemoglobin P-value was (P=0.892) and serum creatinine (P=0.792) which was statistically non-significant whereas serum SGPT value was statistically significant (P=0.03). Correlation of clinical and laboratory parameters with duration of chelation was also estimated. There was no significant effect of duration of chelation therapy on serum ferritin in patients who received chelation for less than 36 months, 56.9% had value above 2500ng/ml, between 37 to 60 months of chelation therapy 50% and for more than 60 months 100% had value above 2500 ng/ml There was no significant effect of duration of chelation on serum creatinine, it was found abnormal in 11.5% of patients who received chelation for 37-60 months, significant effect of chelation therapy was observed on SGPT values, as the duration of chelation increased the number of patients having abnormal SGPT values were also raised, patients receiving chelation therapy for 60 months or more showed deranged SGPT levels in 100% of cases. Between 37-60 months 57% and below 36 months 70% had abnormal values (Table-I).

**Table-I:** Comparison of laboratory parameters with duration of chelation therapy.

Laboratory Parameters													
		Serum ferritin				Serum creatinine				Serum SGPT			
Duration of chelation		1000-2500 ng/l		Above 2500 ng/l		Abnormal		Normal		Abnormal		Normal	
	Less than or equal to 36 months	28	43.1%	37	56.9%	0	0%	67	100%	47	70.1%	20	29.9%
	37-60 months	13	50%	13	50%	3	11.5%	23	88.5%	15	57.7%	11	42.3%
	Above 60 months	0	0%	7	100%	0	0%	7	100%	7	100%	0	0%
Total	100%	41	41.8%	57	58.2%	3	3.0%	97	97%	69	69%	31	31%

Comparison of clinical adverse effects with duration of chelation therapy shows that, below 36 months of optimal chelation abdominal pain was 35%, nausea 21%, vomiting 7%, jaundice, 8% anemia was present in 47% patients whereas above 60 months, abdominal pain was 3%, nausea 5% vomiting 3%. Jaundice 2% and anemia, 5% showing that as the duration of chelation increases the severity of adverse effects decreases (Table-II). None of the clinical and laboratory adverse effects required withdrawal of treatment.

**Table-II:** Comparison of clinical adverse effects with duration of chelation therapy.

		Signs and Symptoms				
Duration of chelation		Abdominal Pain	Nausea	Vomiting	Jaundice	Anemia
	Less than or equal to 36 months	35%	21%	7%	8%	47%
	37-60 months	3%	5%	5%	5%	18%
	Above 60 months	3%	5%	3%	2%	5%
Total	100%	41%	31%	15%	15%	70%

## DISCUSSION

Deferasirox is an effective oral chelator for reducing iron overload in multi-transfused β-thalassemia patients. Iron overload in β-thalassemia major patients is associated with high morbidity and mortality due to complications associated with tissue hemosiderosis. These patients therefore require continuous iron chelation with improve compliance and safety. In recent years multiple iron chelation regimen were used including monotherapy, combined and alternative sequential regimen.^14^,^15^ Deferasirox is used as oral monotherapy due to its prolong half life and selective role in reducing tissue iron of heart and liver, however its efficacy in decreasing high iron overload is unpredictable.^16^,^17^

In the present study also serum ferritin reduced with once oral dose of deferasirox but changes in high values was statistically not significant. On the other hand duration of chelation plays an important role in decline of serum ferritin levels. In earlier studies it was shown that prolong treatment for more than twelve to thirty six months with high doses upto 40mg/kg decreases serum ferritin level significantly,^18^,^19^ in our study there was no significant correlation between duration of chelation and serum ferritin as below thirty six months 56.9% had ferritin levels above 2500ng /ml and 43% less than 2500ng, whereas after sixty months of optimal therapy 41.8% had serum ferritin between 1000-2500ng/ml and 58% showed value above 2500ng/ml, probably due to higher serum ferritin levels at the beginning of chelation or inadequate compliance of the drug. It is also reported that changes in iron burden due to high transfusion requirement and variable gastrointestinal absorption of drug may contribute to variable patient response and require dose adjustments.^20^

Various studies have documented improved clearance with doses between 30-40 mg/kg/day without any adverse effects. In our study deferasirox was given in maximum dose of 40 mg/kg/daywith high serum ferritin levels and in patients who did not responded to low dose but there was no increased incidence of adverse effects. Deferasirox is although well tolerated with high safety profile but most common adverse effcts reported are gastrointestinal disturbances, increase liver enzymes, maculopapular skin rash and elevation of serum creatinine levels. In the present study most frequently observed side effects of deferasirox were gastrointestinal symptoms including abdominal pain 41%, nausea 31%, jaundice was found in 15% respectively. Other recent studies has also shown comparable results.^21^,^22^

Another significant adverse effect documented in the present study was elevated liver enzymes, 69% showed raised SGPT after six months of therapy. Duration of therapy also has significant effect on serum SGPT levels, in the present study number of patients having elevated liver enzymes rises with prolong duration of therapy, below thirty six months 70% had raised SGPT levels whereas above sixty months of chelation all patients showed elevated liver enzymes, other studies also reported raised hepatic enzymes levels with increased duration of therapy.^13^ Serum creatinine in various studies was reported to be elevated with prolomg duration of deferasirox therapy,^23^ however in this study it was raised only in 11.5% patients after thirty six months of chelation. Another significant finding in our study was improvement in gastrointestinal symptoms after thirty six months which is not reported previously however one of the earlier studies has shown improvement in liver enzymes at twenty four to thirty six months of treatment.^24^

Most of the adverse effects reported in the present study were mild and did not require dose adjustment or cessation of therapy except in few patients with elevated liver enzymes, temporary withdrawal of chelation dropped enzymes levels to normal values, other clinical trials have also reported dose adjustment and interruption of therapy in few patients only.^25^

### Limitation of the study

Limitation of the present study were less number of patients, loss for follow-ups and few patients previously on combined therapy of oral deferasirox and parenteral desferral for prolong period who were switched to monotherapy of oral deferasirox probably resulting in data bias, however oral deferasirox showed a better safety profile with good compliance and ability to provide prolong chelation therapy with minimal adverse effects.

## CONCLUSION

Deferasirox is an effective oral chelation agent for β-thalassemia major patients with few adverse effects. The study results shows that deferasirox reduces serum ferritin levels after long term therapy, however changes in serum ferritin values were statistically not significant. Most common adverse effects of the drug were gastrointestinal symptoms including abdominal pain, nausea, vomiting and elevation of liver enzymes. Patients receiving deferasirox should be monitored regularly for timely management of side effects that may occur with long term treatment in order to improve compliance and efficacy.
